# Remote multicomponent intervention for older adults with cognitive impairment in community settings: multisite randomised controlled trial

**DOI:** 10.1093/ageing/afag310

**Published:** 2026-10-27

**Authors:** Geon Young Jang, Sungyang Jo, Ji Yeon Baek, Eunju Lee, Sunghwan Ji, Il-Young Jang, Chan Mi Park

**Affiliations:** Division of Geriatrics, Department of Internal Medicine, Asan Medical Center, University of Ulsan College of Medicine, Songpa-gu, Seoul, Korea (the Republic of); Department of Neurology, Asan Medical Center, University of Ulsan College of Medicine, Songpa-gu, Seoul, Korea (the Republic of); Division of Geriatrics, Department of Internal Medicine, Asan Medical Center, University of Ulsan College of Medicine, Songpa-gu, Seoul, Korea (the Republic of); Division of Geriatrics, Department of Internal Medicine, Asan Medical Center, University of Ulsan College of Medicine, Songpa-gu, Seoul, Korea (the Republic of); Division of Geriatrics, Department of Internal Medicine, Asan Medical Center, University of Ulsan College of Medicine, Songpa-gu, Seoul, Korea (the Republic of); Department of Digital Health, SAIHST, Sungkyunkwan University, Gangnam-gu, Seoul, Korea (the Republic of); Division of Geriatrics, Department of Internal Medicine, Asan Medical Center, University of Ulsan College of Medicine, Songpa-gu, Seoul, Korea (the Republic of); Department of Clinical Research Design and Evaluation, SAIHST, Sungkyunkwan University, Gangnam-gu, Seoul, Korea (the Republic of); PyeongChang Health Center and County Hospital, PyeongChang, Gangwon-do, Korea (the Republic of); Marcus Institute for Aging Research, Hebrew SeniorLife, Harvard Medical School, Boston, MA, USA; Division of Gerontology, Beth Israel Deaconess Medical Center, Harvard Medical School, Boston, MA, USA

**Keywords:** geriatric medicine, cognitive impairment, public health, rural health and medicine, frailty, older people

## Abstract

**Background:**

Cognitive impairment is common among community-dwelling older adults and represents a pragmatic target for community-level intervention. This study evaluated the effectiveness of a scalable, remotely delivered multicomponent intervention for older adults with cognitive impairment, integrated into community and primary care settings.

**Methods:**

This 6-month, parallel-group, randomised controlled trial was performed at two public health centres in South Korea, enrolling adults aged 60–80 years with cognitive impairment and preserved functional independence. Participants were randomly assigned (1:1) to a videoconference-based exercise and nutritional support programme or to usual health promotion services. The primary outcome was change in the Montreal Cognitive Assessment–Korean version (MoCA-K) score at 6 months; secondary outcomes included cognitive, functional, physical performance, frailty and depressive measures at 6 and 12 months.

**Results:**

Among 140 participants (mean age 75.0 years; 60% female), equal numbers were randomised to intervention and control groups. At 6 months, the intervention group showed greater improvement in MoCA-K scores compared to the control group [2.3 points; 95% confidence interval (CI) 1.4–3.1], and the difference was sustained at 12 months (1.1 points; 95% CI 0.3–2.0). The intervention was also associated with improvements in functional status and frailty at both time points. No intervention-related adverse events were reported.

**Conclusions:**

A remotely delivered multicomponent intervention improved cognition, functional independence and frailty in older adults with cognitive impairment identified through community-based screening. These findings support the effectiveness of scalable interventions integrated into community and primary care settings, particularly when access to in-person care is limited.

**Trial Registration:**

Clinical Research Information Service (CRIS), KCT0004547; https://cris.nih.go.kr; registered on 12 December 2019.

## Key Points

- A pragmatic remotely delivered exercise and nutrition intervention improved cognitive, functional and frailty-related outcomes in community-dwelling older adults with cognitive impairment.
- The intervention was implemented using existing community public health resources, supporting its scalability in real-world settings.
- Remotely supervised multidomain interventions may provide a scalable option when access to in-person preventive care is limited.

## Introduction

Cognitive impairment is common among community-dwelling older adults and is associated with increased risks of functional decline, loss of independence and progression to dementia [1, 2]. As populations age, identifying and intervening in early stages of cognitive vulnerability has become a major public health priority [3, 4].

Cognitive impairment in older adults is often multifactorial, arising from potentially reversible neuropsychiatric, social, pharmacological and metabolic contributors [5–7], as well as modifiable lifestyle factors such as physical inactivity and poor nutritional status [8]. Multidomain lifestyle interventions, incorporating physical exercise, nutritional support and cognitive engagement, have shown promise in preventing or delaying cognitive decline [9, 10]. The landmark FINGER study has provided robust evidence supporting the effectiveness of such approaches [11]. Subsequent randomised trials using similar multidomain strategies have reported consistent findings [12, 13]. However, many of these studies have been conducted in controlled research settings with strict diagnostic criteria, potentially limiting their applicability to real-world populations encountered in community and primary care settings.

In practice, older adults identified through community-based screening programmes often present with varying degrees of cognitive decline, reflecting a pragmatic target population for scalable intervention strategies. The COVID-19 pandemic further highlighted the need for accessible and sustainable models of care, as disruptions to in-person services disproportionately affected older adults [14]. In this context, remotely delivered interventions have emerged as a potential approach to maintain continuity of care while overcoming barriers related to mobility, access and resource limitations [15, 16].

To address these gaps, we conducted a randomised controlled trial to evaluate the effectiveness of a remotely delivered exercise and nutrition intervention in community-dwelling older adults with cognitive impairment identified through public health screening. We hypothesised that this pragmatic, community-based multidomain intervention would improve cognitive, functional and frailty-related outcomes.

## Methods

### Study design and population

This parallel-group randomised controlled trial (1:1 allocation) was conducted at two public health centres in South Korea: PyeongChang (rural) and Hanam (urban). The study followed the CONSORT 2025 reporting guidelines and was implemented through a collaboration between an academic medical centre and local public health centres.

Participants were recruited from community-dwelling older adults aged 60–80 years who attended public health centres for cognitive concerns or related services. Eligible individuals were invited to participate in a structured exercise and nutrition programme delivered through the public health centres. Participation was voluntary, and individuals who expressed interest and provided informed consent were enrolled after confirmation of study eligibility.

Eligibility required a Mini-Mental State Examination for Dementia Screening (MMSE-DS) score of 19–26. The MMSE-DS was the national standard cognitive screening tool in South Korea at the time of the study [17]. In this operational context, scores above 26 are generally considered to indicate preserved cognitive function, whereas scores below 19 are typically regarded as indicating more advanced cognitive impairment requiring further evaluation. This intentionally broad range was used to ensure the inclusion of participants with diverse educational backgrounds [18, 19].

Functional independence was confirmed by a Functional Activities Questionnaire (FAQ) score < 2 on all items [20, 21]. Participants were excluded if they had a dementia diagnosis or were receiving treatment with acetylcholinesterase inhibitors or memantine, or had severe physical or medical conditions. Detailed criteria are provided in Appendix 1.

### Patient and public involvement

Public health centre practitioners from the PyeongChang and Hanam government contributed during the intervention planning to ensure feasibility within existing community resources. Their feedback supported the adoption of a single, integrated exercise–nutrition programme to enhance practicality and scalability. Participants were not involved in reporting the study findings.

### Intervention

The intervention was delivered using existing community health centre resources, including trained exercise instructors and dietitians. Participants in the intervention group received a 6-month remote, multicomponent programme consisting of supervised exercise and nutritional support. Because the ability to operate a smartphone was an eligibility requirement, remote participation was enabled at the in-person baseline visit: trained staff helped each participant install the videoconferencing application (Zoom) on their own smartphone and provided hands-on instruction in joining and running the sessions, with additional support during the initial sessions as needed.

Twice-weekly, 60-min sessions were remotely supervised in real time, delivered live via a videoconferencing platform (Zoom) by certified physical trainers who monitored participants throughout each session. Each session included 20 min of aerobic exercise, 20 min of resistance training (using elastic bands) and 20 min of balance training, with intensity progressively increased every 4 weeks. Trainers provided real-time verbal feedback to ensure safety and fidelity.

Additionally, participants received an in-person baseline nutritional assessment and individualised dietary advice from a dietitian and were then instructed to self-administer a protein supplement (Selex®, Maeil Dairies, South Korea) twice daily at home (16 g protein per day). The supplement was delivered 1 month at a time, with the next month’s supply provided only after confirmation that the previous supply had been consumed. Supplement intake was self-reported online at each twice-weekly exercise session and recorded for adherence monitoring. Session attendance was verified using session logs. Detailed intervention protocols are provided in Appendix 2.

The control group received standard health promotion services and routine educational materials from their respective public health centres. Participants in both groups continued their usual medical care during the trial.

### Outcomes and measurements

The primary outcome was the change in the Montreal Cognitive Assessment–Korean version (MoCA-K) score from baseline to 6 months [22]. Secondary outcomes included 12-month change in MoCA-K, as well as changes in the Clinical Dementia Rating–Sum of Boxes (CDR-SB), Geriatric Depression Scale–15 (GDS-15), Short Physical Performance Battery (SPPB), handgrip strength, FAQ and a frailty index at 6 and 12 months [23–26]. Specific components of the frailty index are detailed in Appendix 3.

All assessments were conducted in person at baseline, 6 and 12 months by assessors blinded to group allocation. Cognitive, functional and physical performance measures were administered by trained nurses at the public health centres, who had completed government-certified training in assessing older adults with cognitive impairment, including dementia; the CDR was administered by physicians, also blinded, through semi-structured interviews with participants and informants. Beyond the primary and secondary outcomes, we collected demographic data and assessed body composition, nutritional status, physical activity and sarcopenia. The FAQ was assessed through participant and informant interviews. Detailed measurement protocols, operational definitions and assessment tools are provided in Appendix 4.

Adverse events were assessed throughout the study period. During each intervention session, participants were routinely asked by exercise instructors about the occurrence of falls, musculoskeletal injuries or any other health issues. In addition, participants were encouraged to report any health-related events spontaneously during training sessions or scheduled study visits. Information on adverse events was also collected during follow-up assessments. All reported events were reviewed by the study team, and their potential relationship to the intervention was determined.

### Sample size calculation

Sample size was calculated based on preliminary data from a prior internal pilot study, which showed an average improvement of approximately 4 points in MoCA over 3 months. Assuming a two-sided α of 0.05, 80% power, and a 1:1 allocation ratio, we estimated that 48 participants per group (96 total) were required. Considering an adherence rate of 70%, we set the target sample size at 140 participants. No interim analyses or stopping guidelines were planned.

### Randomisation and blinding

We performed stratified randomisation by age (≥75 vs. <75 years) and sex, without blocking. A computer-generated allocation sequence was prepared by an independent coordinator with no participant contact. Group assignments were concealed in sequentially numbered, opaque, sealed envelopes and opened in order of enrolment at each site. Given the nature of the intervention delivered in public health settings, participants and intervention providers could not be blinded. However, outcome assessors who conducted cognitive and functional evaluations were blinded to group allocation to minimise assessment bias.

### Statistical analysis

All analyses followed the intention-to-treat (ITT) principle. Baseline characteristics were compared between groups using independent *t-*tests for continuous variables and chi-square tests for categorical variables. Adjusted mean differences (AMDs) were estimated using linear mixed-effects models with fixed effects for group, time and their interaction (group × time), adjusting for age, sex, education level and enrolment group. A random intercept for participants was included to account for within-subject correlation. Missing data were handled implicitly under the missing-at-random assumption within the mixed-effects modelling framework; no outcome imputation was performed. Analyses not based on mixed models (e.g. t-tests, chi-square tests) were conducted as complete-case analyses.

Effect sizes and 95% confidence intervals (CIs) were reported for all outcomes. A two-sided *P*-value <.05 was considered statistically significant. All analyses were performed using R (version 4.4.1; R Foundation for Statistical Computing, Vienna, Austria), with the lme4 package for mixed models and the emmeans package for marginal estimates.

### Ethical approval

The study was approved by the Institutional Review Board of Asan Medical Center (IRB No. 2019-1395). Written informed consent was obtained from all participants prior to enrolment.

## Results

### Participant enrolment and characteristics

Of 594 community-dwelling older adults assessed for eligibility, 140 (23.6%) were enrolled and randomised between 11 December 2019 and 30 April 2022, across three enrolment groups at two public health centres. The main reasons for non-enrolment were a diagnosis of dementia or use of dementia medications (acetylcholinesterase inhibitors or memantine; *n* = 219) and inability to use a smartphone (*n* = 182; 30.6% of those screened); 33 eligible individuals declined, corresponding to an uptake of 140/173 (80.9%) among those eligible and approached (Figure 1). Reasons for exclusion were not mutually exclusive.

**Figure 1:**
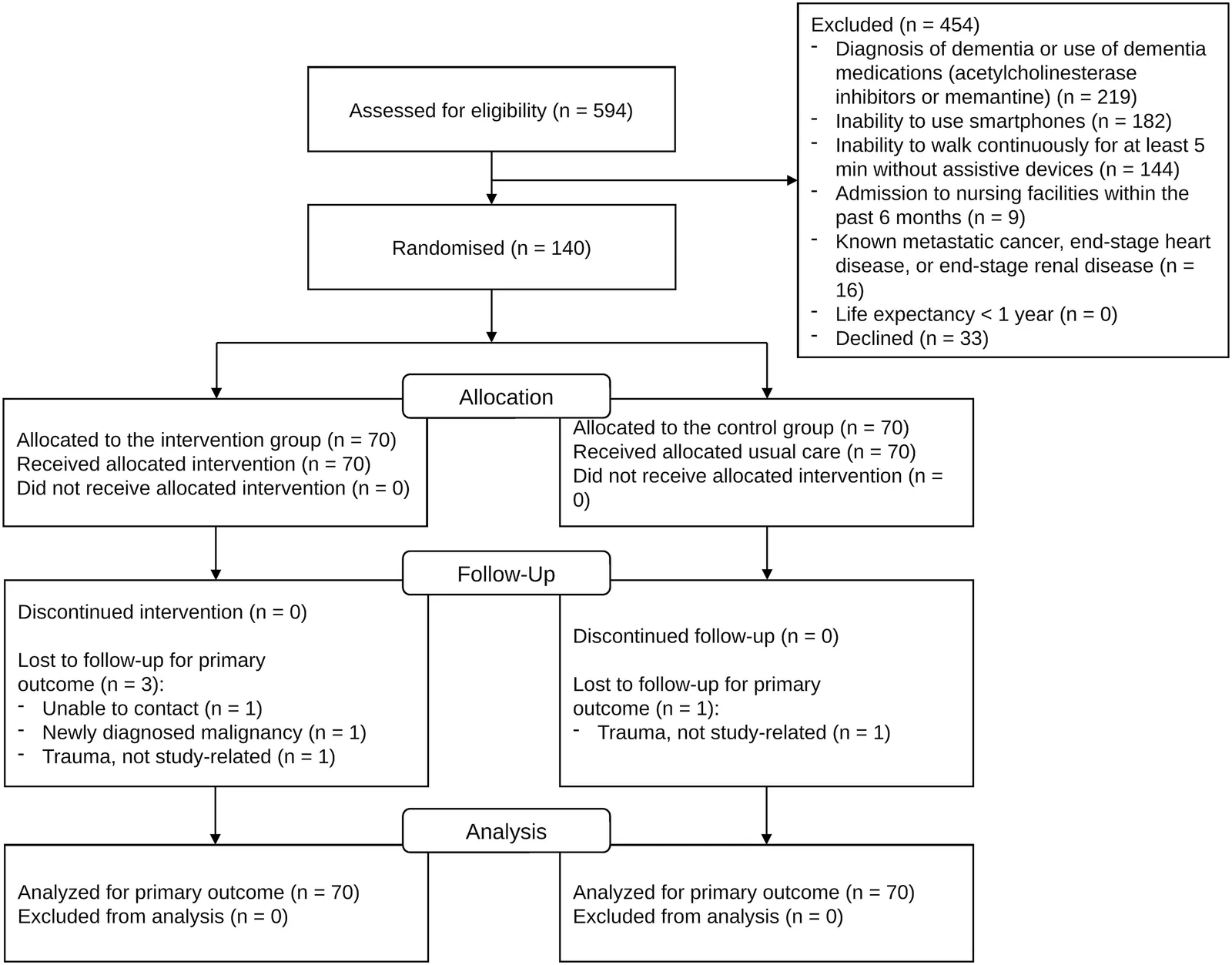
The diagram shows participant eligibility assessment, exclusion, randomisation, follow-up and inclusion in the ITT analysis. Reasons for exclusion were not mutually exclusive.

Details of enrolment and intervention periods by site are presented in Appendix 5. Participants were randomised equally into the intervention (*n* = 70) and control (*n* = 70) groups. At 6 months, the primary outcome assessment was completed by 67 participants in the intervention group and 69 in the control group. At 12 months, 63 intervention and 64 control participants were assessed. All randomised participants were included in the ITT analysis.

Baseline characteristics of the total cohort are summarised in Table 1. The mean age was 75.0 years [standard deviation (SD) 3.8], and 60% (84/140) of participants were female. The majority of participants (85.7%) were living below the relative poverty threshold. No statistically significant differences were observed in baseline characteristics between the intervention and control groups. The mean scores for the total cohort were 19.5 (SD 2.7) for MoCA-K, 23.3 (SD 1.6) for MMSE, 10.2 (SD 1.7) for SPPB, 5.5 (SD 3.3) for GDS-15 and 0.181 (SD 0.085) for the frailty index.

**Table 1:** Baseline characteristics.

Characteristic	Overall (*n* = 140)	Control (*n* = 70)	Intervention (*n* = 70)	*P* value^a^
Age (years)	75.0 (3.8)	75.3 (4.00)	74.7 (3.7)	.38
Sex, female	84 (60.0)	42 (60.0)	42 (60.0)	>.99
Education (years)	7.1 (4.4)	7.7 (4.3)	6.4 (4.4)	.069
Relative poverty	120 (85.7)	57 (81.4)	63 (90.0)	.45
Alcohol consumption, ≥ once per week	18 (12.9)	12 (17.1)	6 (8.6)	.21
Smoking				.88
Never smoker	103 (73.6)	51 (72.9)	52 (74.3)	
Past smoker	26 (18.6)	14 (20.0)	12 (17.1)	
Current smoker	11 (7.9)	5 (7.1)	6 (8.6)	
Chronic disease				
Hypertension	87 (62.1)	45 (64.3)	42 (60.0)	.73
Diabetes mellitus	40 (28.6)	17 (24.3)	23 (32.9)	.35
Dyslipidemia	62 (44.3)	26 (37.1)	36 (51.4)	.13
Myocardial infarct	10 (7.1)	2 (2.9)	8 (11.4)	.10
Stroke	11 (7.9)	6 (8.6)	5 (7.1)	>.99
Sarcopenia	36 (25.9)	15 (21.4)	21 (30.4)	.31
Body mass index	24.9 (3.3)	24.7 (3.2)	25.1 (3.4)	.49
Skeletal muscle index				
Male (*n* = 56)	6.96 (1.30)	7.27 (0.75)	6.65 (1.6)	.075
Female (*n* = 84)	6.10 (0.79)	5.96 (0.69)	6.24 (0.86)	.11
MNA–SF				.41
Normal nutrition	99 (70.7)	48 (68.6)	51 (72.9)	
At risk of malnutrition	37 (26.4)	21 (30.0)	16 (22.9)	
Malnourished	4 (2.9)	1 (1.4)	3 (4.3)	
Low physical activity	37 (26.4)	17 (24.3)	20 (28.6)	.70
MMSE (0–30)	23.3 (1.6)	23.5 (1.4)	23.1 (1.7)	.13
MoCA-K (0–30)	19.5 (2.7)	19.5 (2.7)	19.6 (2.7)	.85
CDR-SB (0–18)	0.65 (0.38)	0.67 (0.39)	0.63 (0.38)	.51
GDS-15 (0–15)	5.5 (3.3)	5.5 (3.1)	5.4 (3.5)	.90
Gait speed (m/s)	0.997 (0.384)	1.001 (0.332)	0.993 (0.432)	>.99
Hand grip strength (kg)				
Male (*n* = 56)	29.3 (9.1)	31.7 (7.6)	26.9 (10.0)	.050
Female (*n* = 84)	19.8 (4.5)	19.1 (4.3)	20.5 (4.7)	.167
SPPB (0–12)	10.2 (1.7)	10.4 (1.4)	10.1 (1.8)	.30
FAQ (0–30)	0.18 (0.45)	0.14 (0.43)	0.21 (0.5)	.35
Frailty index (0–1)	0.181 (0.085)	0.170 (0.075)	0.192 (0.093)	.14

Values are shown as mean (SD) or *n* (%), as appropriate. HNH, Hanam Health Center; PCH, PyeongChang Health Center and County Hospital.

^a^Welch two sample t-test; Pearson’s Chi-squared test.

Follow-up was completed by 136/140 participants (97.1%) at 6 months and 127/140 (90.7%) at 12 months, with similar retention in the intervention (95.7% and 90.0%) and control (98.6% and 91.4%) groups; no participant discontinued because of the intervention. All intervention sessions were delivered via Zoom videoconferencing. Mean adherence to the exercise sessions was 89.3% (PyeongChang) and 72.9% (Hanam) (overall 78.8%), and mean protein supplement intake was 96.1% and 81.7%, respectively (overall 86.9%); the full distribution is provided in Appendix 6. No serious adverse events or intervention-related harms occurred, and reasons for loss to follow-up are detailed in Appendix 7. In a post-intervention satisfaction survey completed by all 70 intervention participants, 94.3% were satisfied with the programme, 95.7% found it easy to understand and follow, 98.6% reported that it helped their health management, and 95.7% wished to continue participating (Appendix 8).

### Outcomes

At 6 months, MoCA-K scores increased from 19.6 (SD 2.7) at baseline to 21.8 (SD 2.6) in the intervention group, whereas scores in the control group remained unchanged [19.5 (SD 2.7) at baseline and 19.5 (SD 3.0) at 6 months] (Table 2). The between-group AMD was 2.3 points (95% CI, 1.4–3.1; *P* < .001; full model specification in Appendix 9). The per-protocol analysis yielded similar findings, with an AMD of 2.14 points (95% CI, 1.27–3.00; *P* < .001) (Appendix 10). MoCA-K scores remained higher at 12 months (AMD, 1.1 points, 95% CI 0.3–2.0, *P* = .009) compared with the control group (Table 2). The longitudinal trajectory is shown in Figure 2A. Results for the MMSE, administered as a supportive measure, are provided in Appendix 11.

**Table 2:** Outcome measures at baseline, end of intervention and end of follow-up.

Variable	Group	Mean (SD)			Change after 6 months (end of intervention)			Change after 12 months (end of follow-up)		
		Baseline	6 months	12 months	AMD (95% CI)^a^	*P* value^b^	Cohen d^c^	AMD (95% CI)^a^	*P* value^b^	Cohen d^c^
MoCA-K (0–30)	Control	19.5 (2.7)	19.5 (3.0)	19.6 (2.6)						
	Intervention	19.6 (2.7)	21.8 (2.6)	20.6 (2.1)	2.3 (1.4, 3.1)	<.001	0.84	1.1 (0.3, 2.0)	.009	0.42
MMSE (0–30)	Control	23.5 (1.4)	22.7 (2.1)	22.9 (1.6)						
	Intervention	23.1 (1.7)	24.9 (1.9)	23.9 (1.7)	2.6 (2.0, 3.3)	<.001	1.70	1.6 (1.0, 2.3)	<.001	1.05
CDR-SB (0–18)	Control	0.67 (0.39)	0.78 (0.49)	0.92 (0.61)						
	Intervention	0.63 (0.38)	0.56 (0.38)	0.57 (0.50)	−0.18 (−0.34, −0.02)	.025	−0.47	−0.32 (−0.48, −0.16)	<.001	−0.83
GDS-15 (0–15)	Control	5.5 (3.2)	6.1 (3.0)	6.6 (3.0)						
	Intervention	5.4 (3.5)	6.2 (2.7)	6.2 (3.0)	0.1 (−0.8, 1.1)	.762	0.04	−0.4 (−1.3, 0.5)	.397	−0.12
SPPB (0–12)	Control	10.4 (1.4)	11.0 (1.8)	11.3 (1.3)						
	Intervention	10.1 (1.7)	10.5 (2.1)	10.3 (2.1)	−0.2 (−0.8, 0.4)	.533	−0.12	−0.6 (−1.3, 0.2)	.129	−0.35
Gait speed	Control	1.001 (0.332)	1.223 (0.440)	1.138 (0.377)						
	Intervention	0.993 (0.432)	1.048 (0.251)	0.998 (0.380)	−0.167 (−0.309, −0.026)	.021	−0.43	−0.124 (−0.268, 0.021)	.094	−0.32
Hand grip strength	Control	24.1 (8.5)	24.9 (7.5)	27.0 (9.1)						
	Intervention	23.1 (7.9)	23.6 (7.5)	24.9 (8.1)	−0.2 (−1.5, 1.0)	.710	−0.03	−1.1 (−2.3, 0.2)	.096	−0.13
FAQ sum (0–30)	Control	0.1 (0.4)	0.9 (1.8)	1.3 (2.2)						
	Intervention	0.2 (0.5)	0.3 (0.8)	0.3 (0.8)	−0.6 (−1.1, −0.1)	.015	−1.31	−1.1 (−1.6, −0.6)	<.001	−2.49
Frailty index (0–1)	Control	0.170 (0.075)	0.169 (0.102)	0.186 (0.102)						
	Intervention	0.192 (0.093)	0.162 (0.086)	0.170 (0.088)	−0.031 (−0.055, −0.008)	.001	−0.37	−0.050 (−0.074, −0.027)	<.001	−0.59

Values are presented as mean (SD).

^a^The reported adjusted mean differences represent the between-group differences (intervention vs control) in outcome changes at each follow-up time point, as derived from the group-by-time interaction terms.

^b^Adjusted mean differences were estimated using linear mixed-effects models including a group-by-time interaction, with fixed effects for age, sex, education level and enrolment group and random intercepts for participants.

^c^Cohen’s *d* was calculated as the adjusted mean difference divided by the pooled baseline SD.

**Figure 2:**
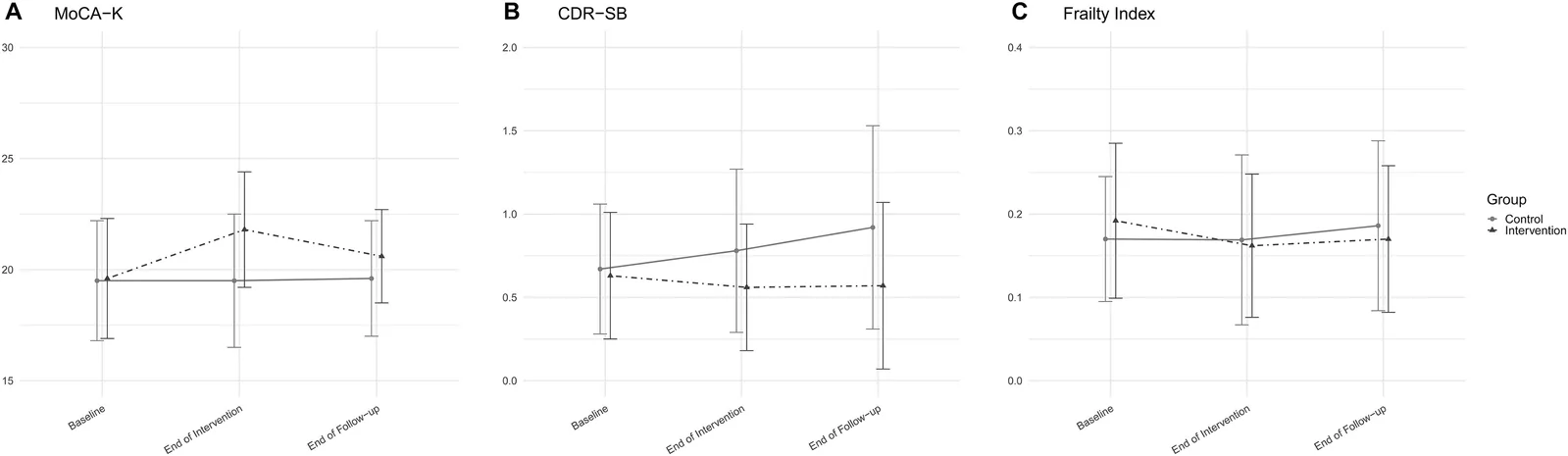
Changes in key cognitive and functional outcomes over time. Each panel shows mean (SD) values at baseline, at the end of intervention (6 months), and at the end of follow-up (12 months) for the intervention and control groups. Outcomes include: (A) MoCA-K. (B) CDR-SB. (C) Frailty index.

Improvements were also observed in the global clinical severity measure, CDR-SB (Figure 2B). For functional and frailty outcomes, FAQ total scores showed AMDs of −0.6 points at 6 months (95% CI −1.1 to −0.1; *P* = .015) and −1.1 points at 12 months (95% CI −1.6 to −0.6; *P* < .001). The frailty index showed AMDs of −0.031 at 6 months (95% CI −0.055 to −0.008; *P* = .001) and −0.050 at 12 months (95% CI −0.074 to −0.027; *P* < .001) (Table 2 and Figure 2C).

During follow-up, a small number of non–intervention-related events were observed in both groups, including trauma, newly diagnosed malignancy and cerebrovascular events. These events occurred outside the intervention context and were not considered to be related to the intervention. No serious adverse events or intervention-related harms were identified.

## Discussion

In this multisite randomised controlled trial, a remotely delivered exercise and nutrition programme was associated with improvements in cognitive, functional and frailty-related outcomes among community-dwelling older adults with cognitive impairment. These benefits were observed over the 6-month intervention period and were partially sustained at 12 months. High adherence and the absence of intervention-related harms support the practical implementation of videoconference-based multidomain interventions within public health settings.

The magnitude of cognitive improvement in this study (MoCA-K + 2.3 points) appears comparable to previous multidomain trials, which nonetheless differ in the populations studied and how the intervention was delivered (Appendix 12). ENHANCE (Peng *et al.*) demonstrated benefits using an intensive, on-site intervention in a relatively higher-functioning, non-demented community population [12]. SUPERBRAIN-MEET (Moon *et al.*) reported cognitive benefits in formally diagnosed Mild Cognitive Impairment (MCI) patients recruited from tertiary-care clinics, using a hybrid model combining recurring in-person facility visits with home-based video sessions and a tablet PC-based application [13]. Two trials delivered interventions fully remotely: Choi *et al.* used a mobile application but relied largely on self-directed engagement, and Li *et al.* delivered a fully remote, real-time supervised programme that was single-domain (exercise only) in a higher-resourced sample [27, 28]. In contrast, our study delivered a supervised, multidomain intervention entirely by real-time videoconferencing to a socioeconomically and educationally vulnerable, community-screened population with lower baseline cognition, a group under-represented in previous multidomain trials. Its contribution therefore lies not in the novelty of the individual components, but in demonstrating that such an intervention can be implemented at scale through existing public health infrastructure for a population that is typically difficult to reach and retain in trials of this kind. The between-group difference in MoCA-K scores exceeded published anchor-based minimal clinically important difference estimates for the MoCA in MCI (approximately 0.7 points), suggesting a clinically relevant effect that persisted, albeit attenuated at 12 months [29].

The observed cognitive benefits may be attributed to the combined effects of physical activity and nutritional support; the structured, real-time group format may additionally have provided opportunities for social interaction, although this was not directly measured and is considered only a possible contextual factor [4, 30–33]. However, given the relatively preserved baseline function of participants and the limited sensitivity of available instruments, changes in physical and mood-related measures were heterogeneous, and their contribution should be interpreted with caution.

Improvements in frailty and functional independence (FAQ) were also observed, with changes appearing to be driven primarily by cognitive improvements rather than consistent gains in physical performance. As the frailty index includes cognitive elements, this should be considered when interpreting the findings. This remains clinically meaningful, as cognitive improvement within a cognitively vulnerable population is a plausible therapeutic response. Although mechanistic inference remains limited, the bidirectional relationship between frailty and cognitive decline supports the plausibility of this finding [34, 35]. Frailty is also a dynamic, potentially modifiable condition and frailty remission has been associated with reduced dementia risk [36].

Although designed before the COVID-19 pandemic, the trial was implemented across multiple sites during periods of social-distancing restrictions. Such pandemic-related social isolation and disruption of daily activities among older adults have been associated with adverse cognitive and mental health outcomes [37, 38]. The majority of participants in this trial had low income levels, reflecting a socioeconomically vulnerable population. Notably, a substantial proportion of participants were at risk of malnutrition on the Mini Nutritional Assessment-Short Form (MNA-SF), indicating meaningful nutritional vulnerability in this population. Such populations are more likely to experience the adverse consequences of pandemic-related disruptions, including greater cognitive decline and more limited access to health and social services [39–41]. Remotely delivered interventions may therefore offer a scalable approach for maintaining cognitive and functional health where in-person access is limited.

This pragmatic trial delivered an integrated exercise–nutrition programme through existing public health resources to a population intentionally defined by community screening rather than formal diagnosis, to reflect the heterogeneous older adults, spanning low educational attainment, mild cognitive impairment and early-stage dementia. This design enhances the relevance and generalisability of the findings but has several limitations. First, diagnostic certainty was limited, as neuroimaging and neuropsychological assessments were not performed and the broad inclusion range introduced heterogeneity, so findings reflect a screening-defined population; the multicomponent design also precluded disentangling the exercise and nutritional effects, and supplement adherence was self-reported. Second, we did not use a double-baseline procedure or alternate forms, so a residual contribution of practice effects to within-group change cannot be excluded; however, the randomised design, in which control-group scores remained essentially unchanged, limits this concern for the between-group comparison, and the MMSE, used only as a supportive measure, should be interpreted with caution given its screening overlap and limited sensitivity to change. Third, social connectedness and loneliness were not directly measured, limiting assessment of psychosocial pathways. Finally, this study was not designed as a formal feasibility trial and did not prespecify formal feasibility thresholds, and generalisability beyond these rural and urban, pandemic-era settings should be interpreted with caution given possible residual confounding by site and time.

## Conclusions

In this pragmatic, multisite randomised controlled trial, a remotely delivered multidomain intervention combining exercise and nutritional support improved cognitive function, frailty status and functional independence among community-dwelling older adults with cognitive impairment. These findings support the effectiveness and scalability of implementing resource-efficient interventions within real-world public health settings.

## Supplementary Material

aa-26-1755-File004_afag310

## Research Data Transparency and Availability

Owing to the sensitive nature of the participant data and restrictions imposed by the approving Institutional Review Board (Asan Medical Center) and the Korean Personal Information Protection Act, the individual participant data cannot be made publicly available. De-identified data may be made available to qualified researchers from the corresponding author on reasonable request, subject to ethics committee approval and a data-sharing agreement. The statistical code is available from the corresponding author on reasonable request.
