# Supplementary material for: Remote multicomponent intervention for older adults with cognitive impairment in community settings: multisite randomised controlled trial
Source: Age Ageing. 2026 Oct 27;55(10):afag310. doi: 10.1093/ageing/afag310 (PMC13710769; doi:10.1093/ageing/afag310)
Supplement: Supplementary file 1 [file aa-26-1755-file004_afag310.docx]

**Remote Multicomponent Intervention for Older Adults with Cognitive Impairment in Community Settings: Multisite Randomised Controlled Trial**

**Supplementary Data**

**Contents**

Appendix 1. Detailed eligibility and exclusion criteria

Appendix 2. Detailed intervention protocols

Appendix 3. Twenty-eight items of the Frailty Index

Appendix 4. Detailed measurement protocols

Appendix 5. Intervention periods by enrolment groups

Appendix 6. Distribution and summary statistics of adherence to exercise sessions and protein supplement intake rate

Appendix 7. Reasons for loss to follow-up at 6 and 12 months

Appendix 8. Post-intervention satisfaction (acceptability) survey in the intervention group

Appendix 9. Adjusted mixed model for MoCA-K score

Appendix 10. Per-protocol outcome measures at baseline, end of intervention, and end of follow-up

Appendix 11. Changes in MMSE at the end of intervention (6 months) and at the end of follow-up (12 months) for the intervention and control groups

Appendix 12. Comparison of remotely delivered multidomain / exercise cognitive intervention trials

Appendix 13. References for supplementary materials

**Appendix 1.** Detailed eligibility and exclusion criteria

| Category | Detailed Criteria |
| --- | --- |
| Inclusion Criteria | - Age 60–80 years - MMSE-DS score: 19–26 points - Functional independence: FAQ score < 2 on all items |
| Exclusion Criteria | - Diagnosis of dementia - Use of acetylcholinesterase inhibitors or memantine - Metastatic cancer End-stage heart disease - Renal disease - Life expectancy < 1 year - Inability to walk for 5 minutes without an assistive device - Inability to own or operate a smartphone - Admission to a nursing facility within the past six months |
| Permitted Medications | - Cognitive enhancers - Ginkgo extract - Choline alfoscerate - and cerebral circulation-improvement agents |

**Appendix 2.** Detailed intervention protocols

A. Detailed Exercise Protocol

| Component | Duration | Examples of Exercises & Progression |
| --- | --- | --- |
| Aerobic | 20 min | Rhythmic routines (Borg RPE 11–14); modified seated routines provided for those with balance issues. |
| Resistance | 20 min | Upper-body (chest, back, arms) and lower-body (quadriceps, gluteal) using elastic bands; 3 sets of 10–15 reps with monthly progression. |
| Balance | 20 min | Static and dynamic postural control (e.g., single-leg stand, tandem walking, multidirectional weight shifting). |

B. Nutritional Profile of the Protein Supplement (per 125 mL serving)

| Nutrient | Amount per serving |
| --- | --- |
| Energy | 135 kcal |
| Sodium | 105 mg |
| Carbohydrate | 15 g |
| └ Sugars | 8 g |
| Protein | 8 g |
| Total Fat | 5 g |
| └ Trans Fat | 0 g |
| └ Saturated Fat | 1.1 g |
| └Cholesterol | 6 mg |
| Calcium | 290 mg |
| Iron | 4 mg |
| Phosphorus | 6 mg |
| Vitamin A | 250 μg RAE |
| Vitamin B1 (Thiamine) | 0.6 mg |
| Vitamin B2 (Riboflavin) | 0.45 mg |
| Niacin | 3.2 mg NE |
| Vitamin B6 | 1.0 mg |
| Vitamin C | 20 mg |
| Vitamin D | 10 μg |
| Vitamin E | 4.6 mg TE |
| Folate | 85 μg DFE |
| Pantothenic Acid | 1.4 mg |
| Biotin | 8 μg |

**Appendix 3.** Twenty-eight items of the Frailty Index

| Financial management | MMSE |
| --- | --- |
| Bill payment and meetings | CDR-SB |
| Shopping | Low muscle mass |
| Recreational activities | Low grip strength |
| Simple food preparation | Chair stand test |
| Meal preparation | Balance test |
| Memory recall | Gait speed test |
| Reading comprehension | Low physical activity |
| Remembering appointments | GDS-15 |
| Transportation/mobility | Exhaustion |
| Hypertension | Mini Nutritional Assessment–Short Form |
| Diabetes mellitus | Weight loss |
| Dyslipidemia | BMI |
| History of myocardial infarction |  |
| History of stroke |  |

Abbreviations: BMI = body mass index, CDR-SB = Clinical Dementia Rating–Sum of Boxes, GDS-15 = Geriatric Depression Scale–15 item, MMSE = Mini-Mental State Examination.

**Appendix 4.** Detailed measurement protocols

| Variable / Instrument | Measurement Protocol / Operational Definition | Reference |
| --- | --- | --- |
| Height, weight | Measured using standard procedures; used to calculate BMI and SMI | — |
| Relative poverty | Monthly household income was assessed by self-report and categorised as below or above 1,000,000 Korean Won (approximately USD 750), corresponding to 50% of the median disposable income for a single-person household in South Korea in 2021. | [1] |
| BMI | BMI = weight (kg) / height² (m²) | — |
| BIA | InBody 620 (InBody Co., Seoul, Korea); multi-frequency BIA; participants barefoot and lightly clothed; used to estimate appendicular skeletal muscle mass (ASM) | — |
| SMI | SMI = ASM (kg) / height² (m²); height-adjusted muscle mass | [2] |
| Handgrip strength | Measured using a dynamometer (T.K.K. 5401 Grip-D, Takei, Tokyo, Japan); two trials with the dominant hand in the standing position; the higher value was used for analysis | [2] |
| SPPB | Standardized assessment of balance, gait speed, and chair stand; total score range 0–12 | [3] |
| Sarcopenia | Defined according to the Asian Working Group for Sarcopenia criteria based on muscle mass and strength | [2] |
| MNA-SF | Standard questionnaire used to assess nutritional status | [4] |
| IPAQ | Standard questionnaire; weekly energy expenditure calculated and expressed as kcal/week | [5] |
| Low physical activity | Defined using IPAQ-derived energy expenditure (<494.65 kcal/week for males and <283.50 kcal/week for females), corresponding to the lowest quintile of total energy expenditure in a population-based Korean survey of older adults. | [6] |
| MoCA-K | Standard administration; primary outcome; change in total score analysed | [7] |
| MMSE | Standard administration; secondary cognitive outcome | [8] |
| CDR-SB | Semi-structured interview-based assessment; change in total score analysed | [9] |
| FAQ | Informant- or participant-based assessment; total score analysed | [5] |
| GDS-15 | Standard questionnaire; total score analysed | [10] |
| FI | Derived from collected data using the deficit accumulation approach; continuous score ranging from 0 to 1; components detailed in Appendix 3 | — |

Abbreviations: ASM = appendicular skeletal muscle mass, BIA = bioelectrical impedance analysis, BMI = body mass index, CDR-SB = Clinical Dementia Rating–Sum of Boxes, FAQ = Functional Activities Questionnaire, FI = frailty index, GDS-15 = Geriatric Depression Scale–15, IPAQ = International Physical Activity Questionnaire, MMSE = Mini-Mental State Examination, MNA-SF = Mini Nutritional Assessment–Short Form, MoCA-K = Montreal Cognitive Assessment–Korean version, SMI = skeletal muscle mass index, SPPB = Short Physical Performance Battery.

**Appendix 5.** Intervention periods by enrolment groups

| Enrolment group | Number of participants | Enrolment period | Intervention period |
| --- | --- | --- | --- |
| PyeongChang Health Center (PCH) | 55 | December 2019 –January 2020 | February–July 2020 |
| Hanam Health Center 1 (HNH1) | 40 | July–September 2020 | October 2020–March 2021 |
| Hanam Health Center 2 (HNH2) | 45 | March–April 2022 | May–October 2022 |

**Appendix 6. Distribution and summary statistics of adherence to exercise sessions and protein supplement intake rate.**

|  | N | Adherence of exercise sessions | Protein supplement intake rate |
| --- | --- | --- | --- |
| HNH | 45 | 72.9 (5.0) | 81.7 (4.8) |
| PCH | 25 | 89.3 (4.8) | 96.1 (4.6) |
| Total | 70 | 78.8 (4.6) | 86.9 (4.6) |

Note: Values are presented as mean (standard deviation).

Abbreviations: HNH = Hanam Health Center, PCH = PyeongChang Health Center and County Hospital

**Appendix 7. Reasons for loss to follow-up at 6 and 12 months**

| Time point | Intervention group | Control group |
| --- | --- | --- |
| 6 months | Unable to contact (n = 1) | Trauma, not study related (n = 1) |
|  | Newly diagnosed malignancy (n = 1) |  |
|  | Trauma, not study related (n = 1) |  |
| 12 months (additional) | Loss to follow-up (unable to contact) (n = 2) | Cognitive decline preventing assessment (n = 2) |
|  | Relocation (n = 1) | Cerebrovascular event (n = 1) |
|  | Death (n = 1) | Trauma (n = 1) |
|  |  | Unable to contact (n = 1) |

**Appendix 8. Post-intervention satisfaction (acceptability) survey in the intervention group.**

| **Survey item** | **Strongly agree ① n (%)** | **Agree ② n (%)** | **Neutral ③ n (%)** | **Disagree ④ n (%)** | **Strongly disagree ⑤ n (%)** | **Favourable ①+② n (%)** |
| --- | --- | --- | --- | --- | --- | --- |
| 1. Overall, how satisfied were you with this health-management service? | 48 (68.6) | 18 (25.7) | 3 (4.3) | 1 (1.4) | 0 (0.0) | **66 (94.3)** |
| 2. Was the content of this health-management service easy to understand and put into practice? | 53 (75.7) | 14 (20.0) | 3 (4.3) | 0 (0.0) | 0 (0.0) | **67 (95.7)** |
| 3. Were you able to feel interest in and engagement with this health-management service? | 53 (75.7) | 14 (20.0) | 3 (4.3) | 0 (0.0) | 0 (0.0) | **67 (95.7)** |
| 4. Did this health-management service help improve your health habits and manage your conditions? | 58 (82.9) | 11 (15.7) | 1 (1.4) | 0 (0.0) | 0 (0.0) | **69 (98.6)** |
| 5. Do you wish to continue participating in this health-management service? | 66 (94.3) | 1 (1.4) | 2 (2.9) | 1 (1.4) | 0 (0.0) | **67 (95.7)** |

*Note: Values are n (%). All 70 intervention-group participants completed the survey (100% response rate). Each item was rated on a 5-point Likert scale (① strongly agree, ② agree, ③ neutral, ④ disagree, ⑤ strongly disagree). “Favourable” denotes a response of strongly agree or agree (① + ②). The control group did not receive the intervention and was not surveyed.*

**Appendix 9.** Adjusted mixed model for MoCA-K score

**A. Fixed Effects**

| Parameter | Estimate | SE | df^*^ | t-value^†^ | *p* value^†^ | 95% CI |
| --- | --- | --- | --- | --- | --- | --- |
| (Intercept) | 22.240 | 3.693 | 128.937 | 6.022 | < .001 | (14.934–29.547) |
| Intervention Effect | 0.283 | 0.430 | 249.201 | 0.658 | .511 | (–0.563–1.129) |
| Time 2 Effect | 0.008 | 0.299 | 257.706 | 0.026 | 0.980 | (–0.581–0.596) |
| Time 3 Effect | –0.179 | 0.307 | 260.310 | –0.585 | 0.559 | (–0.783–0.424) |
| Age (years) | –0.051 | 0.047 | 128.021 | –1.092 | 0.277 | (–0.143–0.041) |
| Sex (Female) | 0.521 | 0.367 | 130.466 | 1.418 | 0.159 | (–0.206–1.248) |
| Education (years) | 0.185 | 0.047 | 138.412 | 3.947 | < 0.001 | (0.092–0.277) |
| Enrolment group (HNH2) | –0.754 | 0.446 | 129.108 | –1.691 | 0.093 | (–1.635–0.128) |
| Enrolment group (PCH) | –1.095 | 0.470 | 130.260 | –2.330 | 0.021 | (–2.025–−0.165) |
| Intervention × Time 2^‡^ | 2.283 | 0.425 | 258.575 | 5.374 | < 0.001 | (1.446–3.119) |
| Intervention × Time 3^‡^ | 1.137 | 0.435 | 261.079 | 2.616 | 0.009 | (0.281–1.994) |

*df = degrees of freedom

†Fixed effects estimates were obtained from a linear mixed-effects model using restricted maximum likelihood (REML), including a group × time interaction and adjusting for baseline covariates.

‡Time 2 and Time 3 indicate the 6- and 12-month visits, respectively.

**B. Random Effects**

| Component | Variance | SD |
| --- | --- | --- |
| Subject (Intercept) | 3.079 | 1.755 |
| Residual | 3.087 | 1.757 |

Random effects include subject-level intercepts.

Abbreviation: CI = confidence interval, HNH1 = Hanam Health Center 1, HNH2 = Hanam Health Center 2, MoCA-K= Montreal Cognitive Assessment–Korean version, PCH = PyeongChang Health Center, SD = standard error, SE = standard error.

**Appendix 10.** Per-protocol outcome measures at baseline, end of intervention, and end of follow-up

| Variable | Group | Mean (SD) | | | Change after 6 months (end of intervention) | | | Change after 12 months (end of follow-up) | | |
| --- | --- | --- | --- | --- | --- | --- | --- | --- | --- | --- |
|  |  | Baseline | 6 months | 12 months | Adjusted mean difference  Mean (95% CI)^*^ | *p* value^*^ | Cohen’s d^†^ | Adjusted mean difference  Mean (95% CI)^*^ | *p* value^*^ | Cohen’s d^†^ |
| MoCA-K^c^ | Control | 19.7 (2.4) | 19.9 (2.5) | 19.6 (2.6) |  |  |  |  |  |  |
|  | Intervention | 19.6 (2.7) | 22.0 (2.5) | 20.6 (2.1) | 2.14 (1.27, 3.00) | < 0.001 | 1.21 | 1.130 (0.263, 1.997) | 0.011 | 0.64 |

* Adjusted mean differences were estimated using linear mixed-effects models including a group-by-time interaction, with fixed effects for age, sex, education level, and enrolment group, and random intercepts for participants.

† Cohen's d was calculated as the adjusted mean difference divided by the pooled baseline standard deviation.

Abbreviation: MoCA-K= Montreal Cognitive Assessment–Korean version.

**Appendix 11. Changes in MMSE at the end of intervention (6 months), and at the end of follow-up (12 months) for the intervention and control groups.**

| Variable | Group | Mean (SD) | | | Change after 6 months  (end of intervention) | | | Change after 12 months  (end of follow-up) | | |
| --- | --- | --- | --- | --- | --- | --- | --- | --- | --- | --- |
|  |  | Baseline | 6 months | 12 months | AMD  (95% CI)^*^ | *p* value^†^ | Cohen d^‡^ | AMD  (95% CI) ^*^ | *p* value^†^ | Cohen d^‡^ |
| MMSE (0–30) | Control | 23.5 (1.4) | 22.7 (2.1) | 22.9 (1.6) |  |  |  |  |  |  |
|  | Intervention | 23.1 (1.7) | 24.9 (1.9) | 23.9 (1.7) | 2.6 (2.0, 3.3) | < 0.001 | 1.70 | 1.6 (1.0, 2.3) | < 0.001 | 1.05 |

Note: Values are presented as mean (standard deviation).

*The reported adjusted mean differences represent the between-group differences (intervention vs control) in outcome changes at each follow-up time point, as derived from the group-by-time interaction terms.

†Adjusted mean differences were estimated using linear mixed-effects models including a group-by-time interaction, with fixed effects for age, sex, education level, and enrolment group, and random intercepts for participants.

‡Cohen’s *d* was calculated as the adjusted mean difference divided by the pooled baseline standard deviation.

Abbreviations: AMD = adjusted mean difference, CI = confidence interval, MMSE = Mini-Mental State Examination

**Appendix 12.** Comparison of remotely delivered multidomain / exercise cognitive intervention trials

| **Study (year, country)** | **Design, N, setting** | **Population** | **Baseline cognition** | **Intervention & duration** | **Delivery mode** | **Results** | **Note** |
| --- | --- | --- | --- | --- | --- | --- | --- |
| SUPERBRAIN-MEET  (Moon et al., 2025, South Korea) | RCT; N = 300 (148:152)  Setting: tertiary hospital (17 tertiary-care outpatient clinics) | MCI, age 60–85  Education 10.7 vs 10.0 yrs (int vs control) | MMSE 26.5 (SD 2.0) vs 26.4 (SD 2.5) | Metabolic/vascular risk monitoring + cognitive training + physical exercise + nutritional education + motivational enhancement vs usual care  24 weeks | Hybrid (in-person + real-time videoconference)  Directly supervised; tablet-PC app | RBANS total scale index +4.17 (95% CI 1.92–6.43) at 24 wk | Formally diagnosed MCI recruited from 17 tertiary hospitals; recurring in-person facility visits every 1–2 weeks. |
| Choi et al.  (2025, South Korea) | RCT; N = 80 (42:38)  Setting: community (3 public dementia relief centres) + secondary general hospital outpatient clinic | MCI with ≥1 modifiable risk factor  ≤6 yrs education: 54.8% (int) / 63.2% (control) | MMSE-KC 23.7 (SD 3.8) vs 22.1 (SD 3.5) | Diet + physical activity + vascular/metabolic monitoring via app vs standard health-care guidelines  52 weeks | Remote, mobile app, home-based  Self-directed (self-logging + asynchronous feedback) | MMSE-KC F = 10.6, p<0.001 at 52 wk | App-based, self-directed (not real-time supervised); recruited via dementia/hospital clinics rather than public-health screening. |
| Li et al.  (2023, USA) | RCT (3-arm); N = 318 (105:107:106)  Setting: community (self-referred volunteers, residential homes) | Self-reported memory decline + CDR ≤0.5, ≥65  61% college-educated, 91% non-Hispanic White | MMSE 27.3/27.2/27.2; MoCA 25.2/25.3/25.3 (enhanced TJQ / standard TJQ / stretching) | Cognitively-enhanced TJQ (motor-cognitive dual-task) vs standard TJQ vs stretching  24 weeks | Fully remote, real-time videoconference  Directly supervised (live instructor), 2×/wk 1 h; no non-exercise control | MoCA +2.8 (98.75% CI 2.1–3.6) vs stretching at 24 wk | Single-domain (exercise/dual-task only); self-referred, higher-resourced US sample, near-normal baseline cognition. |
| **Our study**  **(2026, South Korea)** | RCT; N = 140 (70:70)  Setting: community / public health centre (rural + urban) | Community-dwelling 60–80 with cognitive impairment via public health centre screening  85.7% relative poverty, education 7.1 yrs (SD 4.4) | MoCA-K 19.6 (SD 2.7) vs 19.5 (SD 2.7); MMSE 23.1 (SD 1.7) vs 23.5 (SD 1.4) | Supervised exercise (aerobic/resistance/balance) + fixed-dose protein supplement vs usual health promotion services  6 months | Fully remote, real-time videoconference  Directly supervised by certified trainers; initial in-person assessment only | MoCA-K +2.3 (95% CI 1.4–3.1) at 6 mo | Real-time remote delivery to a low-SES, community-screened, cognitively impaired population. |

Abbreviations: CDR, Clinical Dementia Rating; CI, confidence interval; MCI, mild cognitive impairment; MMSE, Mini-Mental State Examination; MMSE-KC, Korean version of MMSE; MoCA, Montreal Cognitive Assessment; MoCA-K, Korean MoCA; RBANS, Repeatable Battery for the Assessment of Neuropsychological Status; RCT, randomised controlled trial; TJQ, Tai Ji Quan.

**Appendix 13. References for supplementary materials**

1.	Statistics Korea, Financial Supervisory Service, Bank of Korea. Survey on Household Finances and Living Conditions 2021. Daejeon: Statistics Korea, 2022.

2.	Chen LK, Woo J, Assantachai P*, et al.* Asian Working Group for Sarcopenia: 2019 Consensus Update on Sarcopenia Diagnosis and Treatment. J Am Med Dir Assoc 2020; 21: 300-7.e2.

3.	Guralnik JM, Simonsick EM, Ferrucci L*, et al.* A short physical performance battery assessing lower extremity function: association with self-reported disability and prediction of mortality and nursing home admission. J Gerontol 1994; 49: M85-94.

4.	Rubenstein LZ, Harker JO, Salvà A, Guigoz Y, Vellas B. Screening for undernutrition in geriatric practice: developing the short-form mini-nutritional assessment (MNA-SF). J Gerontol A Biol Sci Med Sci 2001; 56: M366-72.

5.	Craig CL, Marshall AL, Sjöström M*, et al.* International physical activity questionnaire: 12-country reliability and validity. Med Sci Sports Exerc 2003; 35: 1381-95.

6.	Won CW. Diagnosis and Management of Frailty in Primary Health Care. Korean J Fam Med 2020; 41: 207-13.

7.	Lee JY, Dong Woo L, Cho SJ*, et al.* Brief screening for mild cognitive impairment in elderly outpatient clinic: validation of the Korean version of the Montreal Cognitive Assessment. J Geriatr Psychiatry Neurol 2008; 21: 104-10.

8.	Kim TH, Jhoo JH, Park JH*, et al.* Korean version of mini mental status examination for dementia screening and its' short form. Psychiatry Investig 2010; 7: 102-8.

9.	O'Bryant SE, Waring SC, Cullum CM*, et al.* Staging dementia using Clinical Dementia Rating Scale Sum of Boxes scores: a Texas Alzheimer's research consortium study. Arch Neurol 2008; 65: 1091-5.

10.	Bae JN, Cho MJ. Development of the Korean version of the Geriatric Depression Scale and its short form among elderly psychiatric patients. Journal of Psychosomatic Research 2004; 57: 297-305.
